# Complete mitochondrial genomes of three *Cichla* species: Annotation, diversity, and phylogenetic insights

**DOI:** 10.1590/1678-4685-GMB-2025-0008

**Published:** 2026-07-24

**Authors:** Diego Ortiz da Silva, Leonardo Carlos Jeronimo Corvalán, Ramilla dos Santos Braga-Ferreira, Cíntia Pelegrineti Targueta, Marcio Roberto Pie, Leo Caetano Fernandes Silva, Rhewter Nunes, Mariana Pires de Campos Telles

**Affiliations:** 1 Universidade Federal de Goiás, Laboratório de Genética e Biodiversidade, Goiânia, GO, Brazil.; 2 Universidade Estadual de Goiás, Instituto Acadêmico de Ciências da Saúde e Biológicas, Laboratório de Bioinformática e Biodiversidade, Iporá, GO, Brazil.; 3 Universidade Federal de Rondonópolis, Instituto de Ciências Exatas e Naturais, Rondonópolis, MT, Brazil.; 4 Universidade Federal de Goiás, Escola de Veterinária e Zootecnia, Hospital Veterinário, Goiânia, GO, Brazil.; 5 Edge Hill University, Biology Department, Ormskirk, United Kingdom.; 6 Instituto Brasileiro do Meio Ambiente e dos Recursos Naturais Renováveis, Centro de Triagem de Animais Silvestres, Goiânia, GO, Brazil.; 7 Pontifícia Universidade Católica de Goiás, Faculdade de Ciências Médicas e da Vida, Goiânia, GO, Brazil.

**Keywords:** Cichlidae, mitogenome, phylogeny, tucunarés

## Abstract

The Cichlidae family, especially the South American genus *Cichla*, is notable for its rapid diversification and ecological impacts following introductions outside its native range. In this study, we first describe the mitogenomes of three *Cichla* species. The mitogenome of *Cichla piquiti* was sequenced from a sample collected at Serra da Mesa Lake, located in the state of Goiás, Brazil. Additionally, the mitogenomes of *Cichla monoculus* and *Cichla temensis* were assembled using public data. The mitogenomes were assembled using NovoPlasty, and comparative analyses were performed, including those of the *Cichla ocellaris* mitogenome data. The mitogenomes ranged from 16,526 bp (*C. monoculus)* to 16,536 bp (*C. piquiti)*, exhibiting a conserved genomic structure with 13 protein-coding genes, 22 tRNAs, and two rRNAs. These mitogenomes were validated by reconstructing phylogenetic relationships within the Cichlinae subfamily. We identified nucleotide composition biases and observed high nucleotide diversity in the D-loop region. Phylogenetic analysis based on complete mitogenome data indicated that *Cichla* species form a clade with *C. ocellaris*, a sister clade to Retroculini. This study provides new mitogenomic insights into *Cichla*, offering valuable genomic resources for species identification and ecological monitoring.

The Cichlidae family, particularly the Cichlinae subfamily, is known for its rapid diversification and speciation, which make it a valuable group for evolutionary studies (López-Fernández et al., 2010). The genus *Cichla*, commonly referred to as tucunarés, is endemic to South America and occurs in the Amazon, Tocantins, and Orinoco River basins, as well as in smaller rivers of the Guianas (Kullander and Efrem, 2006). These species hold high economic value in aquaculture and sport fishing (Kullander and Efrem, 2006). However, the introduction of *Cichla* species into non-native watersheds, driven by commercial interests, has raised significant ecological concerns (Pelicice and Agostinho, 2009). Molecular tools based on mitochondrial genome data (mtDNA) have been utilized to identify and address the ecological impacts of such introductions (Wang et al., 2021). This study aims to sequence and analyze the mtDNA of three *Cichla* species (*C. piquiti*, *C. monoculus*, and *C. temensis*), compare their genomes, assess phylogenetic relationships within the genus and the Cichlinae subfamily, and identify mtDNA regions with potential for the development of DNA barcode markers.

For this purpose, we sequenced one individual of *C. piquiti* collected at Serra da Mesa Lake, located in the state of Goiás, Brazil, with geographical coordinates of longitude -48° 18’ 53.58” and latitude -13° 57’ 23.13”. The DNA was extracted from the muscle tissue using the animal extraction protocol described by Castro et al. (2020). The DNA concentration obtained was 47 ng/µL, as measured with a Qubit High Sensitivity DNA Kit. DNA purity was assessed using Nanodrop. Illumina’s Nextera DNA Flex protocol was used to prepare the genomic libraries, which were indexed using Nextera DNA CD Indexes (Illumina). The fragmentation of the genomic libraries was checked using the Bioanalyzer High Sensitivity DNA kit. Quantification of the libraries was carried out using both Qubit and qPCR, using Qubit High Sensitivity DNA Kit and KAPA SYBR^®^ FAST qPCR Mix, respectively. Sequencing was carried out using the MiSeq^®^ Reagent Kit v3 (600 cycles). For *C. temensis* and *C. monoculus*, we used sequencing data already available in the NCBI SRA database, corresponding to the Brazilian cichlid whole genome sequencing project (PRJEB48774). The accession numbers are ERR10768311 for *C. temensis* and ERR10789871 for *C. monoculus*. The *C. ocellaris* data (complete mitochondrial genome) used for the comparative analyses was obtained from the NCBI Genome database under access number NC_030272.1.

The quality analysis of the sequences generated for *C. piquiti*, as well as the SRA data obtained from NCBI for *C. temensis* and *C. monoculus*, was carried out using the FastQC software. Trimmomatic v0.32 was then used to remove the Illumina adapters and perform quality control with the parameters “SLIDINGWINDOW:4:15” and “MINLEN:100” in paired-end mode. We assembled all the available libraries for *C. monoculus* (n= 10) and *C. temensis* (n= 9) (Table S1). To assemble the mtDNA, we used a seed sequence of the COI gene specific to each species (*C. piquiti*: JN988800.1, *C. monoculus*: JN988799.1, *C. temensis*: FJ440622.1), all using NovoPlasty v. 4.3.1. After assembling the mtDNA, the genomes were annotated. To do this, the assembled genomes were aligned to the *C. ocellaris* reference sequence (NC_030272.1) and annotated using MITOS2 Galaxy WebServer. After annotation, all the genomes were aligned in MAFFT v. 7.

In the comparative analyses for the genus *Cichla*, we used three mtDNA assembled in this study and the complete mtDNA of *C. ocellaris.* We analyzed the bias in nucleotide composition for this genus using the AT-skew and GC-skew metrics (Perna and Kocher, 1995). We also analyzed the Relative Synonymous Codon Usage (RSCU) in MEGA11 v. 11. We then calculated the nucleotide diversity (π) for the genus *Cichla* (interspecific analysis) and for all the assembled mitochondrial genomes of *C. monoculus* and *C. temensis* (intraspecific analysis) in DnaSP v. 6.12.0, with the aim of identifying hotspot regions that could be used to develop barcode primers.

Comparative and evolutionary analyses were carried out for the subfamily Cichlinae (Neotropical cichlids). We used data from 34 complete and annotated mtDNA obtained from the NCBI Genome database (Table S2). We carried out analyses of genomic similarity, collinearity, rearrangements, and inversions of mtDNA regions using Mauve alignment. In addition, we identified the synonymous (Ks) and non-synonymous (Ka) substitutions and calculated the Ka/Ks ratio in DnaSP v. 6.12.0, using data from all the protein-coding genes identified.

Finally, we reconstructed the phylogeny of the Cichlinae subfamily using data from the 13 protein-coding genes, using a maximum likelihood tree with IQ-TREE v. 2.2.0. To identify the most suitable nucleotide model (GTR+F+R4), we used ModelFinder Plus (Kalyaanamoorthy et al., 2017) and applied 1.000 bootstrap replicates. As an outgroup, we used the species *Oreochromis niloticus*, a widely known African cichlid species belonging to another subfamily (Pseudocrenilabrinae). To visualize and plot the resulting phylogenetic tree, we used FigTree v. 1.4.4.

The mtDNA assemblies (circular genomes) of the three species of the genus *Cichla*, based on the *COX1* gene data of each species, were all successfully carried out with average coverage of over 100x. Regarding the size of the mtDNA species of the genus *Cichla* assembled in this study, the genomes ranged from 16,526 bp (*C. monoculus*) to 16,536 bp (*C. piquiti*), with an average of 16,529.3 bp, standard deviation (SD) of 4.73 (Figure 1). The mtDNA structure of *Cichla* showed conservation and maintenance of the number of genes, including 13 protein-coding genes, 22 tRNAs, and two rRNAs, as expected for the mitochondrial genome of fish and previously described for *C. ocellaris* (Lin et al., 2017).

**Figure 1 -:**
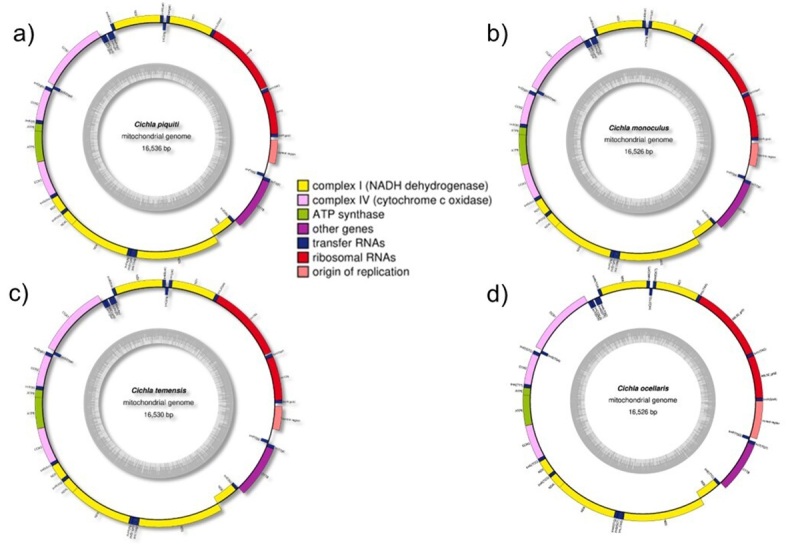
Circular representation of the mtDNA of a) *C. piquiti*; b) *C. monoculus*; c) *C. temensis* and d) *C. ocellaris*. The genes are classified by functional groups, each marked with a distinct color. Inside the larger circle, the genes are transcribed clockwise, while outside this circle, transcription occurs anti-clockwise.

When comparing the complete mtDNA of the genus *Cichla*, the GC content showed an average value of 44.98% (SD = 0.16) (Table S3). Next, the nucleotide composition bias values for the complete genome sequence and *rRNA* showed negative GC-skew values and a positive AT-skew value (Table S3). In this way, we observe a higher presence of C compared to G, and of A compared to T, following the logic of the calculations used for GC-skew ((G - C)/(G+C)) and AT-skew ((A - T)/(A+T)) (Perna and Kocher, 1995). On the other hand, nucleotide composition varies when analyzing the protein-coding genes (PCGs) and *tRNAs*. For the PCG, both the GC-skew and AT-skew values were negative, while for the *tRNAs*, both values were positive (Table S3). Other studies on Teleostei have corroborated these characteristics for the PCG and *tRNAs* (Wang et al., 2023). To explore regional variations along the complete mitochondrial genome, a sliding window analysis was performed (window size: 1,000 bp; step: 500 bp), which revealed an increasing negative asymmetry in GC-skew (ranging from -0.125 to -0.589) along the length of the genome in the four species analyzed, reflecting a predominance of C over G, especially in regions closer to the mtDNA regulatory area (S1). In contrast, AT-skew values oscillated between positive and negative, ranging from 0.333 to -0.0097 in the *Cichla* genus, showing more variable regional patterns (S1). The GC-skew averages obtained by this approach were between -0.317 and -0.352, more negative than the values observed for the whole genome, while the AT-skew averages were between 0.067 and 0.080, close to the global average (S1). This suggests that the sliding window analysis captures local fluctuations that are not visible in the genomic average, reinforcing the differences in composition observed between the functional regions of the mtDNA.

The analysis of the PCGs in *C. piquiti* (11,428 bp), *C. monoculus* (11,433 bp) and *C. temensis* (11,437 bp) revealed that 12 of the 13 PCGs are located in the heavy chain (H), with *ND6* being the only gene in the light chain (L), which is defined as the chain with the highest amount of G + T, characterizing it as the heavy chain, as observed in *C. ocellaris* (Lin et al., 2017). All species had 12 PCGs starting with the canonical ATG codon, except *COX1*, which used the GTG codon, a typical feature of fish mitogenomes (Satoh et al., 2016). As for the stop codons, *C. temensis* and *C. piquiti* showed the same pattern observed in *C. ocellaris*, with seven genes ending in complete stop codons (TAA or TAG) and six with incomplete stop codons, while *C. monoculus* had eight genes with complete stop codons and five with incomplete codons (Table S4).

In the four *Cichla* species analyzed, codons ranged from 5,508 (*C. ocellaris* and *C. monoculus*) to 5,512 (*C. piquiti*), with similar codon usage patterns across species (Figure 2). The most abundant codons varied: CUC (leucine) in *C. piquiti*, CCU (proline) in *C. monoculus* and *C. ocellaris*, and CCC (proline) in *C. temensis*, as noted in other cichlids (Fiteha et al., 2022). Leucine and serine were the most abundant amino acids in all species, contrasting with African cichlids, where leucine and alanine were predominant (Fiteha *et al*., 2022).

**Figure 2 -:**
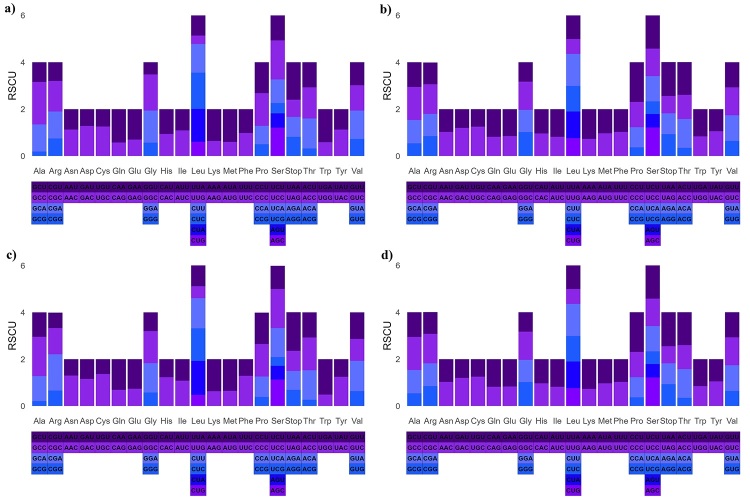
Relative synonymous codon usage (RSCU) analysis for all four species of the genus *Cichla* with complete mitochondrial genomes currently available. a) *C. piquiti*; b) *C. monoculus*, c) *C. temensis* e d) *C. ocellaris*.

Nucleotide diversity in *Cichla* species ranged from 0.012 to 0.104, with the highest values in the D-loop region (Figure 3 A). *NADH dehydrogenase* genes (*ND1*, *ND2, ND4L, ND4*, and *ND5*) frequently showed values above the median. Intraspecific diversity was lower than interspecific diversity. For *C. monoculus*, diversity ranged from 0 to 0.008, peaking in the D-loop, with genes above the median including *COX1*, *COX3*, *ND1*, *ND2*, *ND3*, *ND4*, *ND5*, and *ND6* (Figure S1). For *C. temensis*, diversity ranged from 0 to 0.0025, peaking in *COX1*, with *COX2*, *COX3*, *CytB*, *ATP8*, *ATP6*, *ND1*, *ND2*, *ND3*, *ND4*, *ND4L*, *ND5*, and *ND6* above the median (Figure S2). Regions with high nucleotide diversity are potential targets for designing DNA barcode primers for genus identification and species-specific detection (Wang et al., 2021). The highly variable D-loop region, rich in AT content and microsatellite repeats (Satoh et al., 2016), is particularly useful for population studies and warrants further investigation at individual and species-specific levels. This study may support genomic resources for primer design, addressing the limited availability of nuclear microsatellite markers for *C. piquiti* (Faquim et al., 2022).

**Figure 3 -:**
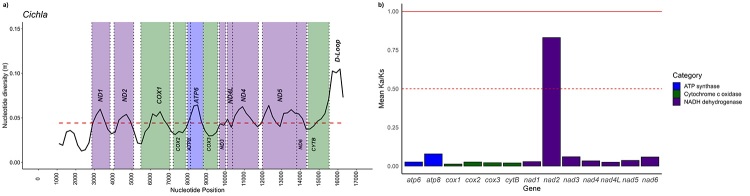
a) Nucleotide diversity (π) calculated for the four species of *Cichla* with mitochondrial genomes assembled in this work and with data available in the NCBI Genome database. The red dashed line represents the median nucleotide diversity, and the peaks above it represent the nucleotide diversity hotspots for the genus. b) Ka/Ks ratios for each of the 13 protein-coding genes estimated for all species in the Cichlinae subfamily. The continuous red line represents the neutral selection value, dashed line represents half of the neutral selection value.

Comparative analyses of the Cichlinae subfamily revealed no genomic rearrangements among 34 species, with the Mauve alignment indicating a single similarity block and high genomic collinearity (Figure S3), consistent with the conserved mtDNA structure in bony fish (Lü et al., 2019). Protein-coding genes showed non-synonymous substitution rates (Ka) ranging from 0 to 0.4045 (mean: 0.0637) and synonymous rates (Ks) from 0 to 1.9992 (mean: 1.199). The highest Ka values were in *NAD2* (0.249) and *ND6* (0.0770), while the highest Ks values were in *NAD5* (1.48) and *NAD1* (1.45), all linked to *NADH dehydrogenase* subunits. The Ka/Ks ratio was calculated to detect selection in the 13 protein-coding genes, all showing values below 1, indicating predominant negative selection (Hurst, 2002). The highest ratio was in *NAD2* (0.831), while *COX1* had the lowest (0.0131). This variation reflects differences in selective pressures across genes (Islam and Sultana, 2022). 

In this study, we present one of the few phylogenetic trees available for the subfamily Cichlinae, based on complete mitochondrial genome data, which includes information from three newly assembled and annotated mitochondrial genomes (Figure 4). The phylogeny, constructed using the maximum likelihood method, supports the monophyletic structure of the subfamily Cichlinae and exhibits a topology consistent with trees derived from morphological data combined with genetic data (Smith et al., 2008; Ilves et al., 2018), as well as those based on mtDNA data (Lao et al., 2023). The target species in this study (*C. piquiti*, *C. monoculus*, *C. temensis* and *C. ocellaris*) were grouped with high support (bootstrap of 100); however, *C. monoculus* and *C. ocellaris* had short branch lengths. In fact, *C. monoculus* and *C. ocellaris* have high morphological and genetic similarity and can be considered evolutionarily significant units within the *C. ocellaris* species (Willis et al., 2012).

**Figure 4 -:**
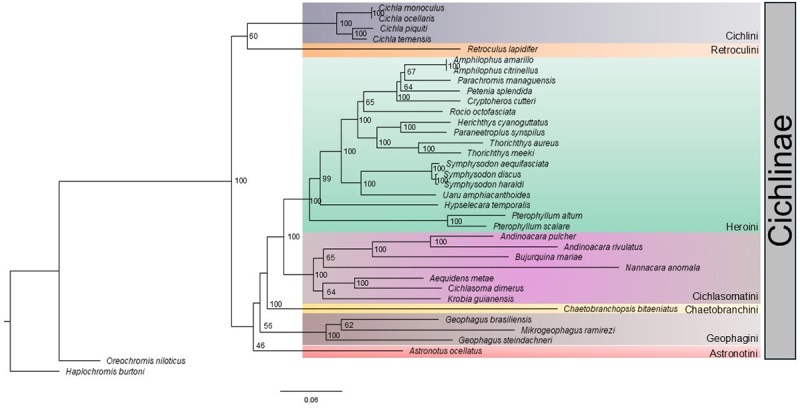
The phylogenetic relationships of the subfamily Cichlinae based on the complete mitochondrial genome. Bootstrap support values with 1,000 replicates are shown at the nodes. *Oreochromis niloticus* was used as the outgroup.

Our phylogeny also indicated that the tribes Cichlini and Retroculini formed a sister group, separating them from the other tribes within the subfamily Cichlinae. Additionally, the tribes Heroini and Cichlasomatini formed a monophyletic group, corroborating the phylogeny based on morphological and genomic data (Smith et al. ,2008; Ilves et al., 2018; Lao et al., 2023). Our results indicated that Astronotini is sister to almost all Cichlinae tribes (except Cichlini and Retroculini), which was also reported by Smith *et al*. (2008). Some node supports are still not well resolved in our phylogeny (<70), suggesting that more genetic resources associated with morphological data are needed to make the phylogeny more robust.

Finally, this study presents three new mitogenomes from the three species (*C. piquiti*, *C. monoculus* and *C. temensis*), which exhibited a structure similar to the mtDNA previously described for *C. ocellaris* and comparable to that of other species in the Cichlinae subfamily. As expected for fish, the mtDNA showed a well-conserved structure, especially regarding gene order and signs of negative selection. The new mtDNA data described here, together with the information on nucleotide diversity, may contribute to future studies, including the development of barcode primers for the rapid identification of these species.

## Supplementary material

The following online material is available for this article:

Table S1 - Sequencing data obtained from the NCBI SRA database for the genus *Cichla*.

Table S2 - Mitochondrial genomes of Neotropical cichlid species obtained from NCBI and used in evolutionary analyses, including sequence ID, GC content percentage, species name, and sequence length.

Table S3 - Nucleotide composition bias and GC content for four *Cichla* mitochondrial genomes.

Table S4 - Description of the mitochondrial protein-coding genes (PCG) for the genus *Cichla*.

Figure S1 - GC and AT skew variation along mitochondrial genomes of four *Cichla* species.

Figure S2 - Nucleotide diversity (π) profiles for *C. monoculus* and *C. temensis* mitogenomes.

Figure S3 - MAUVE progressive alignment showing conserved genomic blocks among Neotropical cichlids.

## Data Availability

 Data used in this study are publicly available in the NCBI databases: *Cichla temensis* (SRA: ERR10768311) and *Cichla monoculus* (SRA: ERR10789871) from project PRJEB48774, *Cichla ocellaris* mitochondrial genome (NC_030272.1), and *Cichla piquiti* mitochondrial genome (NC_084242.1).
