# Supplementary material for: Complete mitochondrial genomes of three Cichla species: Annotation, diversity, and phylogenetic insights
Source: Genet Mol Biol. 2026 Jul 24;49(3):e20250008. doi: 10.1590/1678-4685-GMB-2025-0008 (PMC13403771; doi:10.1590/1678-4685-GMB-2025-0008)
Supplement: Table S1 - [file 1415-4757-GMB-49-3-e20250008-s1.pdf]

Genetics and Molecular Biology
https://doi.org/10.1590/1678-4685-GMB-2025-0008

Supplementary Material to "Complete mitochondrial genomes of three *Cichla* species: Annotation, Diversity, and Phylogenetic Insights"

Table S1 - Sequencing data obtained from the NCBI SRA database for the genus *Cichla*. Information such as the species name, SRA ID, sequencing data and identification of the biological sample are described. The data was obtained from the Brazilian cichlid whole genome sequencing project (PRJEB48774).

Specie	SRA	Information	BioSample
C. monoculus	ERR10789888	run: 80.2M spots, 24.2G bases, 7.4Gb downloads	fCicMon1
C. monoculus	ERR10768224	run: 81.9M spots, 24.7G bases, 7.4Gb downloads	fCicMon2
C. monoculus	ERR10789900	run: 68.4M spots, 20.7G bases, 6.4Gb downloads	fCicMon3
C. monoculus	ERR10789869	run: 56.8M spots, 17.2G bases, 5.2Gb downloads	fCicMon4
C. monoculus	ERR10789885	run: 73.8M spots, 22.3G bases, 6.9Gb downloads	fCicMon5
C. monoculus	ERR10768199	run: 81.1M spots, 24.5G bases, 7.5Gb downloads	fCicMon6
C. monoculus	ERR10789871	run: 82.7M spots, 25G bases, 7.5Gb downloads	fCicMon7
C. monoculus	ERR10768210	run: 53.3M spots, 16.1G bases, 4.9Gb downloads	fCicMon8
C. monoculus	ERR10789866	run: 64.8M spots, 19.6G bases, 5.9Gb downloads	fCicMon9
C. monoculus	ERR10789872	run: 81.1M spots, 24.5G bases, 7.7Gb downloads	fCicMon10
C. temensis	ERR10768276	run: 65.8M spots, 19.9G bases, 6.2Gb downloads	fCicTem1

Specie	SRA	Information	BioSample
C. temensis	ERR10768261	run: 81.4M spots, 24.6G bases, 7.5Gb downloads	fCicTem2
C. temensis	ERR10768311	run: 85.4M spots, 25.8G bases, 8Gb downloads	fCicTem3
C. temensis	ERR10789909	run: 79M spots, 23.9G bases, 7.4Gb downloads	fCicTem4
C. temensis	ERR10768283	run: 68.7M spots, 20.8G bases, 6.4Gb downloads	fCicTem5
C. temensis	ERR10768290	run: 71.3M spots, 21.5G bases, 6.7Gb downloads	fCicTem6
C. temensis	ERR10789855	run: 80M spots, 24.2G bases, 7.3Gb downloads	fCicTem7
C. temensis	ERR10789858	run: 65.4M spots, 19.7G bases, 6Gb downloads	fCicTem8
C. temensis	ERR10789842	run: 70.7M spots, 21.3G bases, 6.4Gb downloads	fCicTem9
