# Supplementary material for: Complete mitochondrial genomes of three Cichla species: Annotation, diversity, and phylogenetic insights
Source: Genet Mol Biol. 2026 Jul 24;49(3):e20250008. doi: 10.1590/1678-4685-GMB-2025-0008 (PMC13403771; doi:10.1590/1678-4685-GMB-2025-0008)
Supplement: Table S2 - [file 1415-4757-GMB-49-3-e20250008-s2.pdf]

Genetics and Molecular Biology
https://doi.org/10.1590/1678-4685-GMB-2025-0008

Supplementary Material to "Complete mitochondrial genomes of three *Cichla* species: Annotation, Diversity, and Phylogenetic Insights"

Table S2 - Mitochondrial genomes of Neotropical cichlid species obtained from NCBI and used in evolutionary analyses, including sequence ID, GC content percentage, species name, and sequence length. *Indicates the mitochondrial genomes assembled in this study, while **denotes the outgroup.

ID	% GC	Description	Sequence Length
NC_033544.1	46.4%	*Aequidens metae*	16541
KY315559.1	45.8%	*Amphilophus amarillo*	16521
KJ081546.1	45.8%	*Amphilophus citrinellus*	16522
NC_033547.1	43.1%	*Andinoacara pulcher*	16513
NC_025671.1	43.1%	*Andinoacara rivulatus*	16585
NC_009058.1	45.0%	*Astronotus ocellatus*	16569
NC_033543.1	41.1%	*Bujurquina mariae*	16540
NC_033542.1	41.5%	*Chaetobranchopsis bitaeniatus*	16610
NC_084243.1	45.6%	*Cichla monoculus**	16526
NC_030272.1	45.6%	*Cichla ocellaris*	16526
NC_084242.1	45.6%	*Cichla piquiti**	16536
NC_084244.1	45.9%	*Cichla temensis**	16530
NC_033551.1	45.5%	*Cichlasoma dimerus*	16617
NC_033552.1	47.0%	*Cryptoheros cutteri*	16528
NC_031181.1	45.8%	*Geophagus brasiliensis*	16559

ID	% GC	Description	Sequence Length
NC_033545.1	46.1%	*Geophagus steindachneri*	16594
NC_033546.1	46.5%	*Herichthys cyanoguttatus*	16540
NC_011168.1	46.1%	*Hypselecara temporalis*	16544
NC_031440.1	45.7%	*Krobia guianensis*	16539
NC_031439.1	44.6%	*Mikrogeophagus ramirezi*	16526
NC_031183.1	46.6%	*Nannacara anomala*	16502
MT437356.1	46.6%	*Oreochromis niloticus***	16631
NC_026918.1	46.4%	*Parachromis managuensis*	16526
NC_023526.1	47.5%	*Paraneetroplus synspilus*	16543
NC_024835.1	46.8%	*Petenia splendida*	16518
NC_028723.1	45.8%	*Pterophyllum altum*	16495
NC_026535.1	45.8%	*Pterophyllum scalare*	16491
NC_033549.1	47.2%	*Retroculus lapidifer*	16537
NC_033548.1	45.6%	*Rocio octofasciata*	16539
NC_028182.1	45.1%	*Symphysodon aequifasciata*	16545
NC_026689.1	45.1%	*Symphysodon discus*	16544
NC_027965.1	45.1%	*Symphysodon haraldi*	16543
NC_031182.1	47.9%	*Thorichthys aureus*	16530
MZ427899.1	46.8%	*Thorichthys meeki*	16527
NC_033550.1	45.5%	*Uaru amphiacanthoides*	16549
