# Supplementary material for: Complete mitochondrial genomes of three Cichla species: Annotation, diversity, and phylogenetic insights
Source: Genet Mol Biol. 2026 Jul 24;49(3):e20250008. doi: 10.1590/1678-4685-GMB-2025-0008 (PMC13403771; doi:10.1590/1678-4685-GMB-2025-0008)
Supplement: Table S3 - [file 1415-4757-GMB-49-3-e20250008-s3.pdf]

Genetics and Molecular Biology
https://doi.org/10.1590/1678-4685-GMB-2025-0008

Supplementary Material to "Complete mitochondrial genomes of three *Cichla* species: Annotation, Diversity, and Phylogenetic Insights"

Table S3 - Nucleotide composition bias and GC content for four *Cichla* mitochondrial genomes. GC%: Percentage of guanine and cytosine; PCG: Protein coding gene; tRNA genes: transfer RNA genes; rRNA genes: ribosomal RNA genes.

Species	Genome			PCG			tRNA genes			rRNA genes		
	GC%	GC-skew	AT-skew	GC%	GC-skew	AT-skew	GC%	GC-skew	AT-skew	GC%	GC-skew	AT-skew
C. monoculus	44.87	-0.119	0.031	46.77	-0.359	-0.023	43.55	0.034	0.044	46.68	-0.133	0.232
C. ocellaris	44.92	-0.118	0.031	46.80	-0.359	-0.023	43.61	0.036	0.043	46.78	-0.139	0.235
C. piquiti	44.90	-0.131	0.032	46.68	-0.371	-0.018	43.64	0.019	0.042	46.91	-0.126	0.230
C. temensis	45.21	-0.130	0.037	46.84	-0.373	-0.013	44.06	0.023	0.047	46.98	-0.135	0.238
Average	44.98	-0.125	0.033	46.77	-0.366	-0.019	43.71	0.028	0.044	46.84	-0.133	0.234
Standard deviation	0.16	0.007	0.002	0.06	0.007	0.004	0.23	0.008	0.001	0.13	0.005	0.003
