# Supplementary material for: Complete mitochondrial genomes of three Cichla species: Annotation, diversity, and phylogenetic insights
Source: Genet Mol Biol. 2026 Jul 24;49(3):e20250008. doi: 10.1590/1678-4685-GMB-2025-0008 (PMC13403771; doi:10.1590/1678-4685-GMB-2025-0008)
Supplement: Table S4 - [file 1415-4757-GMB-49-3-e20250008-s4.pdf]

Genetics and Molecular Biology
https://doi.org/10.1590/1678-4685-GMB-2025-0008

Supplementary Material to "Complete mitochondrial genomes of three *Cichla* species: Annotation, Diversity, and Phylogenetic Insights"

Table S4 - Description of the mitochondrial protein-coding genes (PCG) for the genus *Cichla*. For each gene, the size in base pairs, the start codon and the stop codon are provided.

Specie	Gene name	Size	Start codon	Stop codon
C. monoculus	NADH dehydrogenase subunit 1	975	ATG	TAA
C. monoculus	NADH dehydrogenase subunit 2	1045	ATG	T
C. monoculus	cytochrome c oxidase subunit I	1563	GTG	TAA
C. monoculus	cytochrome c oxidase subunit II	691	ATG	T
C. monoculus	ATP synthase subunit 8	168	ATG	TAA
C. monoculus	ATP synthase subunit 6	684	ATG	TAA
C. monoculus	cytochrome c oxidase subunit III	785	ATG	TA
C. monoculus	NADH dehydrogenase subunit 3	349	ATG	T
C. monoculus	NADH dehydrogenase subunit 4L	297	ATG	TAA
C. monoculus	NADH dehydrogenase subunit 4	1378	ATG	T
C. monoculus	NADH dehydrogenase subunit 5	1839	ATG	TAA
C. monoculus	NADH dehydrogenase subunit 6	522	ATG	TAG

Specie	Gene name	Size	Start codon	Stop codon
C. monoculus	cytochrome b	1137	ATG	TAA
C. temensis	NADH dehydrogenase subunit 1	975	ATG	TAA
C. temensis	NADH dehydrogenase subunit 2	1045	ATG	T
C. temensis	cytochrome c oxidase subunit I	1563	GTG	TAA
C. temensis	cytochrome c oxidase subunit II	691	ATG	T
C. temensis	ATP synthase subunit 8	168	ATG	TAA
C. temensis	ATP synthase subunit 6	684	ATG	TAA
C. temensis	cytochrome c oxidase subunit III	785	ATG	TA
C. temensis	NADH dehydrogenase subunit 3	349	ATG	T
C. temensis	NADH dehydrogenase subunit 4L	297	ATG	TAA
C. temensis	NADH dehydrogenase subunit 4	1378	ATG	T
C. temensis	NADH dehydrogenase subunit 5	1839	ATG	TAA
C. temensis	NADH dehydrogenase subunit 6	522	ATG	TAG
C. temensis	cytochrome b	1141	ATG	T
C. piquiti	NADH dehydrogenase subunit 1	975	ATG	TAA
C. piquiti	NADH dehydrogenase subunit 2	1045	ATG	T
C. piquiti	cytochrome c oxidase subunit I	1554	GTG	TAG
C. piquiti	cytochrome c oxidase subunit II	691	ATG	T
C. piquiti	ATP synthase subunit 8	168	ATG	TAA
C. piquiti	ATP synthase subunit 6	684	ATG	TAA

Specie	Gene name	Size	Start codon	Stop codon
C. piquiti	cytochrome c oxidase subunit III	785	ATG	T
C. piquiti	NADH dehydrogenase subunit 3	349	ATG	T
C. piquiti	NADH dehydrogenase subunit 4L	297	ATG	TAA
C. piquiti	NADH dehydrogenase subunit 4	1378	ATG	T
C. piquiti	NADH dehydrogenase subunit 5	1839	ATG	TAA
C. piquiti	NADH dehydrogenase subunit 6	522	ATG	TAA
C. piquiti	cytochrome b	1141	ATG	T
C. ocellaris	NADH dehydrogenase subunit 1	975	ATG	TAA
C. ocellaris	NADH dehydrogenase subunit 2	1045	ATG	T
C. ocellaris	cytochrome c oxidase subunit I	1563	GTG	TAA
C. ocellaris	cytochrome c oxidase subunit II	691	ATG	T
C. ocellaris	ATP synthase subunit 8	168	ATG	TAA
C. ocellaris	ATP synthase subunit 6	684	ATG	TAA
C. ocellaris	cytochrome c oxidase subunit III	784	ATG	T
C. ocellaris	NADH dehydrogenase subunit 3	349	ATG	T
C. ocellaris	NADH dehydrogenase subunit 4L	297	ATG	TAA
C. ocellaris	NADH dehydrogenase subunit 4	1378	ATG	T
C. ocellaris	NADH dehydrogenase subunit 5	1839	ATG	TAA
C. ocellaris	NADH dehydrogenase subunit 6	522	ATG	TAG
C. ocellaris	cytochrome b	1137	ATG	TAA
