# Supplementary material for: Complete mitochondrial genomes of three Cichla species: Annotation, diversity, and phylogenetic insights
Source: Genet Mol Biol. 2026 Jul 24;49(3):e20250008. doi: 10.1590/1678-4685-GMB-2025-0008 (PMC13403771; doi:10.1590/1678-4685-GMB-2025-0008)
Supplement: Figure S3 - [file 1415-4757-GMB-49-3-e20250008-s7.pdf]

Genetics and Molecular Biology
https://doi.org/10.1590/1678-4685-GMB-2025-0008

Supplementary Material to "Complete mitochondrial genomes of three *Cichla* species: Annotation, Diversity, and Phylogenetic Insights"

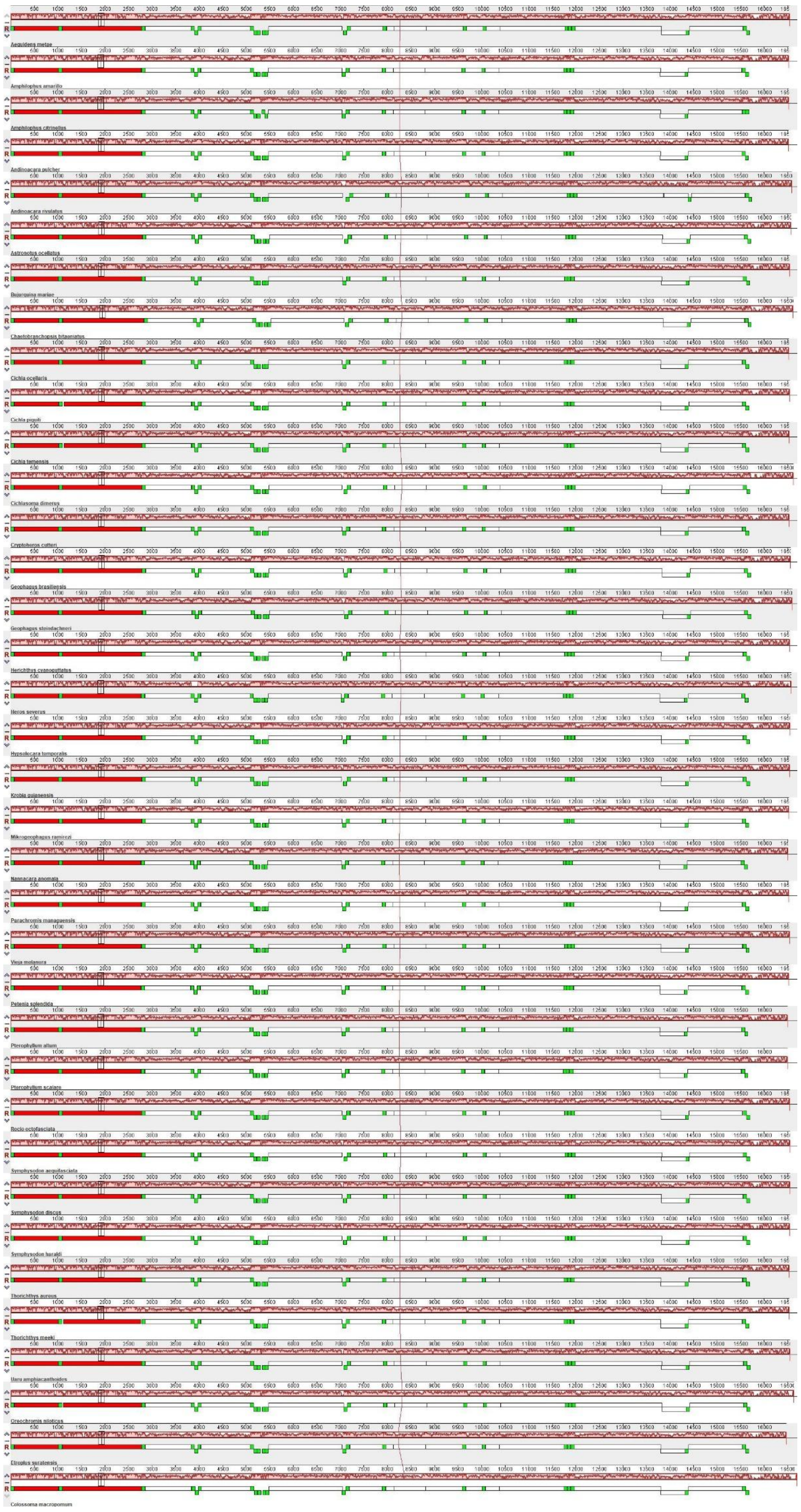

Figure S3 - MAUVE progressive alignment showing conserved genomic blocks among Neotropical cichlids.
